# Venomics of the Central European Myrmicine Ants *Myrmica rubra* and *Myrmica ruginodis*

**DOI:** 10.3390/toxins14050358

**Published:** 2022-05-21

**Authors:** Sabine Hurka, Karina Brinkrolf, Rabia Özbek, Frank Förster, André Billion, John Heep, Thomas Timm, Günter Lochnit, Andreas Vilcinskas, Tim Lüddecke

**Affiliations:** 1 Institute for Insect Biotechnology, Justus Liebig University Giessen, Heinrich-Buff-Ring 26-32, 35392 Giessen, Germany; sabine.hurka@innere.med.uni-giessen.de (S.H.); john.heep@web.de (J.H.); andreas.vilcinskas@ime.fraunhofer.de (A.V.); 2 LOEWE Centre for Translational Biodiversity Genomics (LOEWE-TBG), Senckenberganlage 25, 60325 Frankfurt, Germany; 3 Bioinformatics and Systems Biology, Justus Liebig University Giessen, Heinrich-Buff-Ring 58, 35392 Giessen, Germany; karina.brinkrolf@bio.uni-giessen.de (K.B.); frank.foerster@computational.bio.uni-giessen.de (F.F.); 4 Department of Bioresources, Fraunhofer Institute for Molecular Biology and Applied Ecology, Ohlebergsweg 12, 35392 Giessen, Germany; ozbekrabia@gmail.com (R.Ö.); andre.billion@ime.fraunhofer.de (A.B.); 5 Institute of Biochemistry, Justus Liebig University Giessen, Friedrichstraße 24, 35392 Giessen, Germany; thomas.timm@biochemie.med.uni-giessen.de (T.T.); guenter.lochnit@biochemie.med.uni-giessen.de (G.L.)

**Keywords:** insect venom, proteotranscriptomics, biodiscovery, allergens, phospholipase A1, EGF-like toxins

## Abstract

Animal venoms are a rich source of novel biomolecules with potential applications in medicine and agriculture. Ants are one of the most species-rich lineages of venomous animals. However, only a fraction of their biodiversity has been studied so far. Here, we investigated the venom components of two myrmicine (subfamily Myrmicinae) ants: *Myrmica rubra* and *Myrmica ruginodis*. We applied a venomics workflow based on proteotranscriptomics and found that the venoms of both species are composed of several protein classes, including venom serine proteases, cysteine-rich secretory protein, antigen 5 and pathogenesis-related 1 (CAP) superfamily proteins, Kunitz-type serine protease inhibitors and venom acid phosphatases. Several of these protein classes are known venom allergens, and for the first time we detected phospholipase A1 in the venom of *M. ruginodis*. We also identified two novel epidermal growth factor (EGF) family toxins in the *M. ruginodis* venom proteome and an array of additional EGF-like toxins in the venom gland transcriptomes of both species. These are similar to known toxins from the related myrmicine ant, *Manica rubida*, and the myrmecine (subfamily Myrmeciinae) Australian red bulldog ant *Myrmecia gullosa*, and are possibly deployed as weapons in defensive scenarios or to subdue prey. Our work suggests that *M.* *rubra* and *M. ruginodis* venoms contain many enzymes and other high-molecular-weight proteins that cause cell damage. Nevertheless, the presence of EGF-like toxins suggests that myrmicine ants have also recruited smaller peptide components into their venom arsenal. Although little is known about the bioactivity and function of EGF-like toxins, their presence in myrmicine and myrmecine ants suggests they play a key role in the venom systems of the superfamily Formicoidea. Our work adds to the emerging picture of ant venoms as a source of novel bioactive molecules and highlights the need to incorporate such taxa in future venom bioprospecting programs.

## 1. Introduction

Ants (Formicidae) are a species-rich family within the hyperdiverse insect order Hymenoptera. They emerged in the Cretaceous about 130 million years ago, and have diversified into more than 14,000 species distributed across 17 subfamilies [1,2]. They have conquered all continents and represent arguably one of the most successful lineages among terrestrial animals [3]. Although most ant species are found in the tropics, remarkable diversity is also present in more temperate regions [1]. For example, 108 genera are native to central Europe, most of which are members of the subfamily Myrmicinae (52% of European ant species at time of writing) [1]. The evolutionary success of ants is based upon an unprecedented array of ecological innovations, such as their eusociality, foraging strategies [3], and the venom systems present in several ant subfamilies [4].

Venoms are complex mixtures of proteins, peptides and organic molecules, and are found throughout the animal kingdom [5]. Venomous animals inject venom into other animals to disrupt important physiological processes [6]. The detrimental effects of venom mainly reflect the activities of proteins and peptides collectively described as toxins. Across all animal groups, venom serves the three major functions of predation, defense and competitor deterrence [6], as well as many secondary functions, such as sexual communication, prey tracking and maintenance of the immune system [7]. Venom toxins have been subject to millions of years of natural selection, becoming refined molecular weapons that act with unmatched selectivity and potency [5]. The venom system of ants is found at the anterior end of the abdomen and is derived from an ancient ovipositor [8]. Ants primarily use their venoms to overpower prey and defend their colony against threats [4]. Ant venoms are mainly composed of polypeptides and small organic molecules, particularly alkaloids [4,9,10,11].

Although there are several venomous ant lineages, only a few ant venoms have been studied in detail [12,13,14,15,16,17,18]. This is primarily because small arthropods deliver only miniscule amounts of venom, so it may be necessary to sample hundreds of specimens to accumulate sufficient amounts of crude venom for traditional analytical platforms [4,19,20]. This challenge has been addressed by the new research field of venomics, where modern systems biology approaches are used to disentangle venom components, often by combining proteomics and transcriptomics in an approach known as proteotranscriptomics [21]. Given the specificity and sensitivity of such modern platforms, small samples are sufficient for analysis and venoms can now be explored across the entire animal kingdom [21].

The application of venomics provides valuable insights into the chemical diversity of venoms from many underrepresented groups of venomous animals, especially arthropods, but ant venoms have still been largely overlooked [22]. Only a handful of proteotranscriptomics studies have been conducted on ant venoms, so a significant knowledge gap remains [23,24,25,26,27]. Furthermore, the studies conducted thus far have mostly focused on enigmatic species such as the bullet ant (*Paraponera clavata*) and the red bulldog ant (*Myrmecia gullosa*), two atypically large ant species with potent nociceptive venoms [15,22]. Proteotranscriptomics has also been applied to *Manica rubida*, one of the larger species of myrmicine ants from central Europe [14]. Beyond these prominent species, there has been little progress in the investigation of ant venoms using modern approaches.

To fill this important knowledge gap, we investigated the venoms of *Myrmica rubra* and *Myrmica ruginodis*, two smaller myrmicine ants that are ubiquitous in central Europe [1]. We applied our modern venomics workflow to identify the venom components, including novel toxins of the epidermal growth factor (EGF)-like family. We discuss our findings in relation to data available from other ant species, including *M. rubida*. This work advances our understanding of ant venoms and can be used as the basis for further studies on the venomics of ants and other arthropod lineages.

## 2. Results

### 2.1. Verification of Species Identity

We conducted initial morphological examinations to ensure that the ant specimens were correctly identified. We measured the diameter of the scapus base and the scapus length–head width ratio to distinguish *M. rubra* and *M. ruginodis* from other Myrmicinae [25]. We also considered features such as the petiolus shape, postpetiolus posture and propodeal spines to differentiate between *M. rubra* and *M. ruginodis*. As a second line of evidence, we retrieved the mitochondrial *cytochrome oxidase 1* (*CO1*) gene, a common barcoding gene for animals (Supplementary Tables S1 and S2) and compared them to available barcodes for species of Formicidae. The retrieved *CO1* sequences matched published *M. rubra* and *M. ruginodis* barcodes, thus validating our morphological examination.

### 2.2. Proteomic and Transcriptomic Landscapes

**Figure 1:**
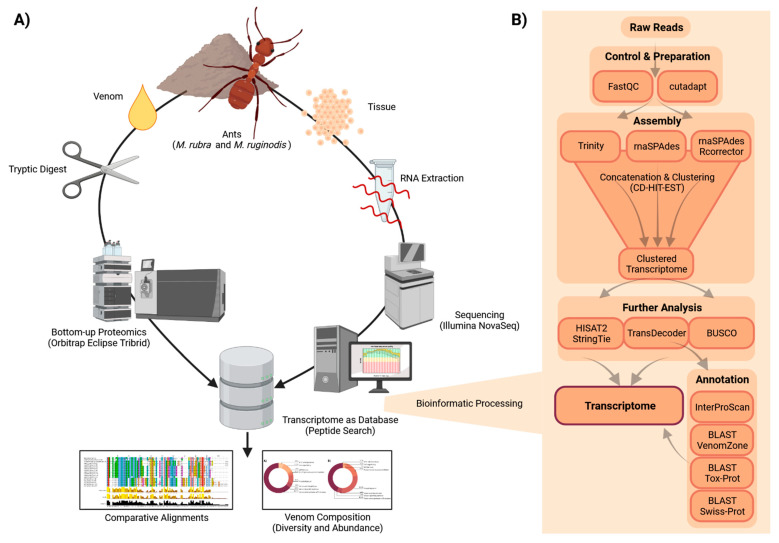
Overview of our proteotranscriptomics workflow. (**A**) Proteomics workflow: Crude venom was collected, digested and analyzed in a bottom–up proteomics approach using an Orbitrap Eclipse Tribrid MS and the transcriptome database. (**B**) Transcriptomics workflow: RNA was sequenced on an Illumina NovaSeq system, and the raw sequencing data were preprocessed and assembled using multiple algorithms. The concatenated dataset was further analyzed and annotated based on different sources of information. The resulting ORFs were used as a database for the proteomics experiment. Transcripts validated at the proteome level were used for the subsequent analysis of venom components in both species.

We used our bespoke proteotranscriptomics workflow (Figure 1) to investigate the venom components of both ant species. This generated 69,929,174 sequencing reads for *M. rubra* and 69,617,668 for *M. ruginodis* (69,645,529 and 69,318,655, respectively, after trimming and quality control). The clustered assemblies contained 512,855 (*M. rubra*) and 466,158 (*M. ruginodis*) unique contigs. We mapped the trimmed reads onto the two assemblies, revealing minimum mapping rates of 96.30%. BUSCO results showed that at least 94.2% of the expected hymenopteran genes were present (*M. rubra* C:95.5% [S:8.8%, D:86.7%], F:1.6%, M:2.9%, n:5991; *M. ruginodis* C:94.2% [S:8.8%, D:85.4%], F:2.0%, M:3.8%, n:5991).

De novo transcriptomes for venom glands are known to overestimate the diversity of toxin transcripts and produce false-positive sequences [28,29,30]. This phenomenon is likely to be amplified by the use of multiple assemblers. Therefore, we added proteomic verification to our analysis and used our clustered assembly as a database for peptide searches. For the peptide searches, Mascot assigned the spectra to 2085 (*M. rubra*) and 1695 (*M. ruginodis*) different open reading frames (ORFs). We removed 1797 (*M. rubra*) and 1314 (*M. ruginodis*) spectra from the dataset using our filtering protocol (Section 5.6). All remaining hits were covered by at least two peptides. When combining data from both species, the molecular weight of venom components ranged from 2.6 to 377.8 kDa, with sequence lengths of 21–3392 amino acids. We annotated 255 of the 288 candidates in *M. rubra* and 348 of the 381 candidates in *M. ruginodis* using BLAST.

### 2.3. Venom Composition of Myrmica rubra

We identified 44 proteins in *M. rubra* venom with similarities to known venom proteins, and these were assigned to three protein families. The vast majority (37/44 or ~84% of total protein diversity) belonged to the venom serine protease or S1 protease family (hereafter VSPs). Another four (~9%) belonged to the venom acid phosphatase family, and the remaining three (~7%) were related to the CAP superfamily (cysteine-rich secretory proteins, antigen 5 and pathogenesis-related 1 proteins). VSPs were not only the most diverse protein family among the *M. rubra* venom candidates but also the most abundant. The sum of all VSP members expressed in transcripts per million (TPM) was 76.7% of all contigs in the *M. rubra* venom gland, followed by the CAP proteins (18.5%) and the venom acid phosphatases (4.8%).

### 2.4. Venom Composition of Myrmica ruginodis

**Figure 2:**
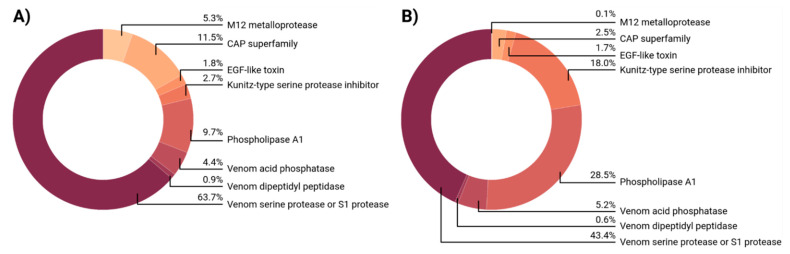
Venom composition of *M. ruginodis* by venom protein family. (**A**) Protein diversity based on absolute numbers of represented sequences. (**B**) Abundance based on transcripts per million (TPM).

We identified 113 proteins in *M. ruginodis* venom with similarities to known venom proteins, and these were assigned to eight protein families (Figure 2). VSPs were again the most diverse (72/113, or ~64% of total protein diversity), followed by CAP proteins (13/113, 11.5%) and phospholipase A1 (PLA) (11/113, ~10%). Six proteins (~5%) were assigned to the M12 metalloproteases, five (~4%) to the venom acid phosphatases, and three (~3%) to the Kunitz-type serine protease inhibitors. Another two proteins (~2%) were members of the EGF-like family of toxins, and one (~1%) was a putative S9 dipeptidyl peptidase. VSPs were again the most abundant components, accounting for 43.4% of all transcripts in the *M. ruginodis* venom glad. The PLAs were next (28.5%), followed by Kunitz-type serine protease inhibitors (18.0%), and the remainder were minor components: CAP proteins (2.5%), EGF-like toxins (1.7%) and venom dipeptidyl peptidase (0.6%). The least abundant protein family was the M12 metalloprotease, accounting for 0.1% of all transcripts in the *M. ruginodis* venom gland.

### 2.5. Diversity and Characteristics of EGF-Like Toxins in Myrmicine Ants

The venom proteome of *M. ruginodis* contained two members of the EGF-like toxin family, whereas none were found in the *M. rubra* proteome. We therefore interrogated the transcriptomes of both species for additional EGF-like toxins by performing a BLAST search against the transcriptomic datasets. We identified three additional EGF-like toxin precursors in *M. ruginodis* (making five in total) and four such sequences in *M. rubra*. SignalP analysis and alignments with known ant EGF-like toxins revealed that these are secreted proteins that are expressed as prepeptides with an adjacent signal peptide but no propeptide between the EGF-like domain and the signal peptide. The predicted mature toxins were 49–56 amino acids in length with predicted molecular masses of 5.2–6.0 kDa (Table 1). All corresponding contigs showed higher TPM values or were tracked only in samples from venom gland tissue and not in body tissue samples.

To gain further insight into the diversity and function of ant EGF-like toxins, we analyzed their similarity to related sequences in silico. We compared the mature sequences of all putative EGF-like toxins in both species to all known EGF-like toxins from other ants. This alignment revealed an overall similar architecture, featuring a conserved six-cysteine backbone and several conserved sites, particularly within the signal peptide. The propeptide, found in plesiotypic and nontoxic EGF hormones, was not present in the EGF-like toxins. Despite the overall similarity among the sequences, we observed some differences in the inter-cysteine spacing and length.

We constructed a maximum likelihood phylogenetic tree to find unrecognized differences between the proteins and to gain insight into their evolutionary history and relationship. The tree contained three major clades of EGF-like toxins, each exclusively comprising toxins from one subfamily of ants. The MYRTX-clade contained all EGF-like toxins from Myrmicinae (taxonomically represented by *M. rubra, M. ruginodis* and *M. rubida*) and achieved 77% bootstrap support. The MIITX-clade was classified as a sister group of the myrmicine toxins and contained EGF-like toxins from Myrmeciinae (*M. gulosa* and *Myrmecia chrysogaster*). It received 97% bootstrap support. Finally, the ECTX-clade was classified as a sister group to all remaining ant EGF-like toxins, and comprised ectatommine toxins found only in *Rhytidoponera metallica*. It also received 100% bootstrap support. Although EGF-like toxins from Myrmeciinae and Ectatomminae formed a single clade and are thus similar at the sequence level, a much higher degree of sequence diversity was indicated for members of the MYRTX-clade. Here, three potential subclades were identified which we named MYRTX-clades A, B and C. MYRTX-clade A contains four EGF-like toxins identified in *M. ruginodis* and two identified in *M. rubra* (U-MYRTX-Mrub1a). MYRTX-clade B has a single member belonging to *M. rubra* (U-MYRTX-Mrub1c). MYRTX-clade C contains the remaining EGF-like toxins from *M. rubra* and *M. ruginodis*, and U18-MYRTX-Mri1a contains the remaining EGF-like toxins from *M. rubida*. However, some of these shallow clades within the MYRTX lineage received only marginal bootstrap support values and the relationships between them must be interpreted with caution.

Finally, we employed a strategy related to that of Eagles and colleagues [31] by initiating a BLAST search to identify patterns of similarity between ant EGF-like toxins and known EGF hormones across the animal kingdom (Table 2). We found that five major subgroups of EGF-like toxins exist in ants. Interestingly, the similarity subgroups revealed by this approach corresponded to clades within the EGF-like toxin phylogeny. The first similarity subgroup comprised the two peptides in the MIITX-clade, which are highly similar to vertebrate heparin-binding EGF (HBEGF) hormones. The second similarity subgroup comprised the members of the ECTX-clade, which are similar to vertebrate betacellulin and epiregulin. The remaining three similarity subgroups featured EGF-like toxins from the MYRTX-clade (myrmicine ants). The most diverse of these three groups contained EGF-like toxins resembling vertebrate transforming growth factor α (TGFα) hormones and comprised six members: two from *M. rubra* (U-MYRTX-Mrub1a and U-MYRTX-Mrub1d) and four from *M. ruginodis* (U-MYRTX-Mrug1a, 1b, 1d and 1e), representing MYRTX-clade A. The second group contained a single peptide (U-MYRTX-Mrub1c) related to vertebrate epiregulin, corresponding to MYRTX-clade B. The remaining three EGF-like toxins (U18-MYRTX-Mri1a, U-MYRTX-Mrub1b and U-MYRTX-Mrug1c) resemble insect Spitz-like proteins and mostly represent MYRTX-clade C.

## 3. Discussion

### 3.1. Serine Proteases and Kunitz-Type Serine Protease Inhibitors

**Figure 3:**
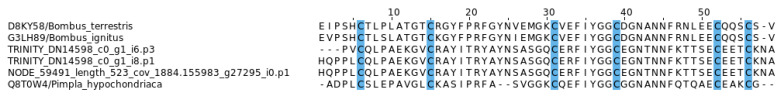
Sequence similarities between Kunitz-type serine protease inhibitors from *M. ruginodis* and other hymenopteran species (*Bombus terrestrix*, *Bombus ignitus* and *Pimpla hypochondriaca*). Conserved cysteine residues are shown in blue.

VSPs dominated the venom profile of both *M. rubra* and *M. ruginodis* and share similarities with known members of this class from other hymenopteran genera, particularly *Apis*, *Bombus* and *Polistes*. Like other serine proteases, VSPs hydrolyze peptide bonds and cause protein degradation, often triggering cytotoxic or hemotoxic effects [32]. However, few VSPs from insect venom have been analyzed in detail. One exception is Bi-VSP from the venom of *Bombus ignites*, which is similar to several of the *M. rubra* and *M. ruginodis* VSPs. Bi-VSP is a multifunctional serine protease that activates the phenoloxidase cascade and causes lethal melanization in insects, but in mammalian blood it has coagulotoxic effects by activating prothrombin and cleaving fibrinogen [33]. Given the similarity between Bi-VSP and several VSPs in *M. rubra* and *M. ruginodis*, it is possible that at least some of the latter possess similar insecticidal and coagulotoxic activities. Interestingly, Bi-VSP acts in concert with a co-injected Kunitz-type serine protease inhibitor (Bi-KTI), which inhibits plasmin as part of a multipronged attack on the coagulation cascade [34]. Although we found no evidence for the presence of Kunitz-type serine protease inhibitors in *M. rubra* venom at the proteomic level, three members of this class were present in the venom proteome of *M. ruginodis*, and they were the third most abundant component in this sample based on transcriptome data. Sequence comparisons revealed that Kunitz-type serine protease inhibitors in myrmicine ants are closely related to those found in the parasitoid wasp *Pimpla hypochondriaca* (UniProtKB Q8T0W4) and the bumblebee *Bombus terrestris* (Figure 3) [35]. In particular, the *B. terrestris* protein (Bt-KTI, UniProtKB D8KY58) is similar to Bi-KTI (UniProtKB G3LH89) from *Bombus ignitus* and can likewise inhibit plasmin [36]. It is therefore possible that the combinatorial mode of action between VSPs and Kunitz-type serine protease inhibitors observed in *B. ignitus* venom may also be prevalent in myrmicine ants [34]. However, the venom profiles of other myrmicine ants must be resolved and complemented with bioactivity studies to confirm whether the venom components work in this cooperative manner.

### 3.2. Venom Allergens 

The CAP family is another important group of venom components in *M. rubra* and *M. ruginodis*, although particularly in the latter, where they contribute significantly to the venom profile. CAP proteins are found in most animal venoms and are functionally very diverse [6,37]. For example, cone snail CAP proteins may function as proteolytic enzymes, whereas snake CAP proteins are neurotoxins [6]. In ants and other hymenopterans, CAP proteins are clinically relevant because they are major allergens that underlie the reactions to bee stings and ant bites [38]. We identified three CAP proteins in the venom of *M. rubra* and 13 in *M. ruginodis*. Interestingly, most were highly similar to allergen 3 from the black imported fire ant (*Solenopsis richteri*), another myrmicine species. The CAP allergens in *Solenopsis* venoms can cause severe, sometimes fatal anaphylaxis in humans and are highly cross-reactive [10,39,40,41].

Another important group of hymenopteran venom allergens is the PLAs, which hydrolyze phosphatidylcholine and cause inflammation as well as local tissue damage. Some PLAs are described collectively as allergen 1 and exacerbate the allergic response to ant bites. The analysis of *M. ruginodis* venom in the 1960s revealed a surprising lack of PLAs despite their presence in other hymenopteran venoms [42], leading to predictions that future studies would reveal their presence also in *M. ruginodis* [43]. As predicted, we can report for the first time that PLA is also present in *M. ruginodis* venom, and indeed this is the first ever description of PLAs in this species. We identified 11 PLAs, accounting for 18.5% of the transcripts in the venom gland and thus representing the second most abundant protein family in *M. ruginodis* venom. Some of these proteins resembled a PLA from *Dinoponera quadriceps*, a ponerine ant, but most showed greater similarity to Sol i 1, an allergen from *Solenopsis invicta*, and are therefore also likely to trigger allergic reactions. 

In summary, potential venom allergens representing the CAP and PLA families are present in the venoms of *M. rubra* and *M. ruginodis*. The candidates resemble proteins from related species that induce allergic reactions and sometimes severe anaphylaxis. In Germany, neither *M. rubra* nor *M. ruginodis* are known to cause significant allergic reactions, although both species are common and synanthropic [44]. However, dangerous allergic reactions have been reported in the USA, where *M. rubra* is considered an invasive pest [45]. Therefore, although *M. rubra* and *M. ruginodis* are not dangerous per se, stings may lead to occasional emergencies and should be treated with caution by the affected individual.

### 3.3. EGF-Like Toxins

The 113 venom components in *M. ruginodis* included two peptides with an EGF-like motif. These form part of a diverse family of metazoan peptide hormones that are also venom components in anemones and ants [14,22,31,46,47]. EGF-like peptides therefore provide another example of the “toxipotent” nature of peptide hormones and their potential to become weaponized as venom components [48,49,50,51,52,53].

Although we identified only two EGF-like peptides in the *M. ruginodis* proteome, the transcripts were among the top 30 most abundant. Given that not all mRNAs are constantly translated into proteins, we screened the venom gland transcriptomes of *M. rubra* and *M. ruginodis* for additional EGF-like toxins that were not detected in the proteome. This revealed four *M. rubra* toxins (U-MYRTX-Mrub1a, 1b, 1c and 1d) as well as three additional toxins from *M. ruginodis* (U-MYRTX-Mrug1c, 1d and 1e). This is a key finding that supports the general but lineage-specific presence of EGF-like toxins in formicoid ant venoms. Several EGF-like toxins have recently been described in the ant subfamilies Myrmeciinae (*M. gullosa* and *M. chrysogaster*), Ectatomminae (*R. metallica*), Myrmicinae (*Myrmica sulconodus, M. rubra, M. ruginodis, M. rubida, Pogonomyrmex californicus* and *Pogonomyrmex barbatus*) and Formicinae (*Formica aquilonia*) [14,22,31]. With the exception of the three peptides from *M. gulosa, M. chrysogaster* and *M. rubida*, all the EGF-like toxins were retrieved from whole-body transcriptomes or genomes, so it is unclear if they are translated into venom proteins [31]. In our study, we found that nine EGF-like toxins were expressed in the venom gland, two of which were also detected at the proteomic level. This suggests that EGF-like toxins are important venom components in Myrmicinae, and supports their presence in other formicoid venoms. However, transcriptome data may overestimate toxin diversity [30] and only two of the nine EGF-like toxins were verified at the protein level. Therefore, we recommend that future studies investigate the presence of EGF-like toxins in the venoms of different ants at the proteome level.

### 3.4. Evolution and Function of Venom Proteins in Myrmicine Ants

Animal venoms have evolved to serve three main functions: predation, defense and competitor deterrence [7]. Although ants use their venom systems mainly to overpower prey and defend their colony, defensive venoms largely underpin their evolutionary success [4]. The eusocial lifestyle of many ant species, with large numbers of individuals forming a single colony, presents a conspicuous target for predators [4]. Vulnerability thus led to the evolutionary innovation of chemical defense systems in ants, with two principal forms: the spraying of formic acid and the injection of venom. Although the acid-based defense system is better known to the general public, the number of species that deploy this mechanism is limited, whereas ~75% of all ant species inject venom [22,54]. Across the animal kingdom, defensive venoms are characterized by their ability to induce pain [55,56,57].

In agreement with this common role of ant venoms, the functional annotation of venom components in *M. rubra* and *M. ruginodis* suggests activities that may facilitate defense. For example, several protein families are associated with proteolytic and/or tissue-damaging activities, including VSPs, M12 metalloproteases and PLAs, which contribute to the painful effect of envenomation. Furthermore, the cooperative attack on the coagulation cascade by VSPs and Kunitz-type serine protease inhibitors may cause local edema and thus additional hypersensitivity.

**Figure 4:**
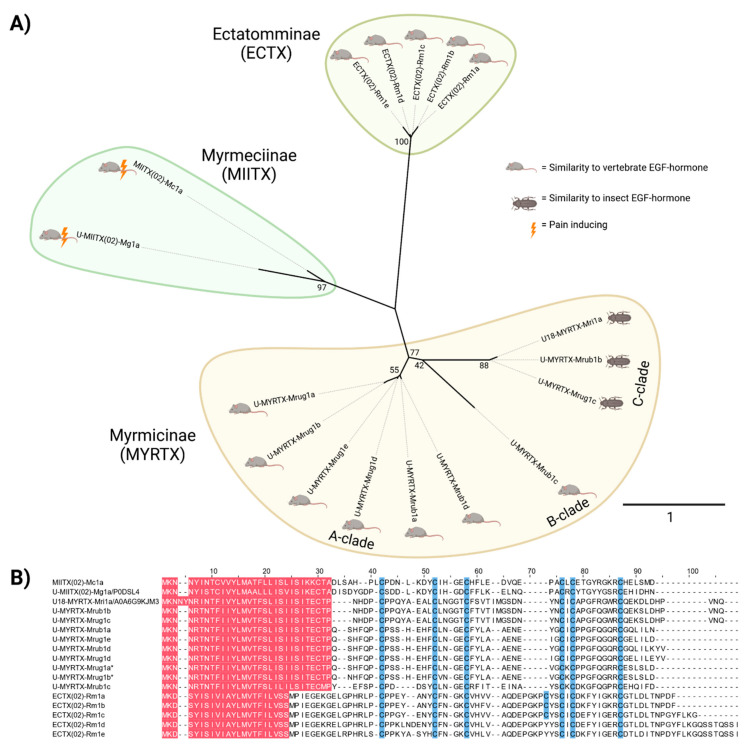
Relationships between all known EGF-like toxins from ants. (**A**) The unrooted phylogenetic tree includes node numbers indicating bootstrap support. The colored boxes indicate the taxonomic placement of the species in which the corresponding toxin was identified. Major clades within the myrmicine EGF-like toxins are indicated (A-clade, B-clade and C-clade). Animal images show the predicted target of each toxin. (**B**) The sequence alignment shows predicted signal peptides (adjusted SignalP prediction and information from UniProtKB) in red and conserved cysteine residues in blue. Toxins marked with an * were discovered in this study.

The most interesting components identified in the venom systems of *M. rubra* and *M. ruginodis* were the EGF-like toxins, whose biological functions remain unclear. The corresponding myrmecine peptides (MIITX-clade) were recently identified as members of this venom protein family [31]. They showed no activity against insects but induced pain in mice for several days [31]. The MIITX EGF-like toxins may therefore have evolved to mimic HBEGF hormones, resulting in their remarkable algogenic effects by activating mammalian ErbB receptors. The authors also found that the other known ant EGF-like peptides resembled either vertebrate betacellulin or insect EGF-like hormones from *R. metallica*, depending on the analysis method. In contrast, the EGF-like toxin previously described in *M. rubida* was most similar to insect Spitz-like proteins, and may therefore have evolved to target insects. Our sequence analysis was largely consistent with these results, annotating the *R. metallica* (ECTX-clade) peptides as betacellulin or epiregulin, the MYRTX-clade C peptides as insect Spitz-like proteins and the *Myrmecia* peptides (MIITX-clade) as HBEGF mimics. Phylogenetic and sequence similarity analysis for the MYRTX peptides indicated a potentially diverse evolutionary history and molecular diversification. Here, functionally divergent peptides may have emerged within the Myrmicinae that share similarity with different vertebrate and insect templates (Figure 4). However, some of the internal branches of the phylogeny were only weakly supported by bootstraps and thus must be interpreted with caution. Future studies adding more EGF-like toxins from other Formicoidea and in particular from the Myrmicinae will help to clarify the relationships and strengthen the proposed hypotheses. That said, our phylogenetic tree suggests that the EGF-like toxins resembling insect Spitz-like proteins (MYRTX-clade C) were more recently recruited to the venom system of myrmicine ants, probably as weapons against other insects given their similarity to insect hormones. MYRTX-clade A appears to be derived from weaponized TGF hormones and may therefore target the corresponding vertebrate receptors, whereas the single peptide representing MYRTX-clade B is strikingly similar to vertebrate epiregulin and is also likely to target vertebrates. It shares this similarity with two peptides from the distant ECTX-clade, suggesting that convergent evolution has resulted in the recruitment of epiregulin into ant venom systems twice, once each in the subfamilies Ectatomminae and Myrmicinae (Figure 4).

Many of the identified EGF-like toxins in the MYRTX-clade may target vertebrates and thus probably act as defensive weapons. This agrees with earlier reports of painful stings (by European standards) and significant neurotoxicity caused by *M. ruginodis*. However, some EGF-like toxins have different effects depending on the type of test [31], so additional analysis with more sophisticated software and a wider range of ant venoms is recommended before drawing firm conclusions. The ability of MYRTX-clades A and B to affect vertebrates could also be studied by isolating or synthesizing them, followed by careful functional assays.

In addition to defense, ant venom can be used for predation and minor roles such as communication, which must be considered when assigning functions to venom components. In *M. rubra* and *M. ruginodis*, the EGF-like toxins of MYRTX-clade C that resemble insect Spitz-like proteins may be used to overpower insect prey, perhaps in cooperation with VSPs. Indeed, many ant venom components may serve multiple functions depending on the context. For example, the universal molecular function of VSPs is the hydrolysis of peptide bonds, but this may fulfil various biological roles including trophic agents when injected to insect prey, spreading factors that facilitate the rapid uptake of co-injected toxins, the decomposition of killed prey to provide nutrition, and the triggering of edema and localized pain in predators. The strict division of venom components into unique functional categories may not be possible, and it may be more valuable to interpret venom exochemistry in a context-dependent manner. Indeed, the multifunctional nature of venom components has been reported for several venomous animals, including some ants [49,58,59,60,61].

## 4. Conclusions

Venoms have contributed to the evolutionary success of many animal lineages, particularly arthropods. Ants are among the most abundant arthropod species, but many have not been studied in detail and questions remain about their evolutionary ecology and biochemistry, including the composition of their venom systems.

We applied a modern venomics workflow based on proteotranscriptomics to shed light on the venom composition of *M. rubra* and *M. ruginodis*, two common myrmicine ant species native to central Europe. We identified several protein families commonly found in ant venoms, including VSPs, Kunitz-type serine proteases, CAP superfamily protein and PLAs. Many of these components are proteolytic enzymes that may be used for predation, defense, as spreading factors, for external digestion or combinations of the above. We also identified several allergenic components in these venoms. Although neither *M. rubra* nor *M. ruginodis* is recognized as medically significant, potentially dangerous anaphylactic shock may occur following envenomation. The previous analysis of *M. ruginodis* venom revealed the conspicuous absence of phospholipases. However, by identifying several members of the PLA family, we demonstrate the presence of these important allergenic substances in *M. ruginodis* venom for the first time. We also detected previously unknown EGF-like toxins in the venom systems of *M. rubra* and *M. ruginodis,* highlighting their importance as lineage-specific ant toxins.

Our work represents a valuable contribution to the growing body of knowledge on the composition, evolutionary ecology and biochemistry of ant venom systems, and we have discussed the potential functions and interactions of the venom components. To test our hypotheses, it will be necessary to isolate or synthesize individual venom peptides and proteins for bioactivity assays. It is also important to broaden the taxonomic coverage of ant venomics, not only to include fellow species of Myrmicinae but also other subfamilies. Such studies may also lead to the discovery of novel bioactive components that can be translated into drugs and bioinsecticides.

## 5. Materials and Methods

### 5.1. Animals

Specimens were collected from two colonies each of *M. rubra* and *M. ruginodis* in Giessen (Hesse, Germany). The ants were kept in plastic containers (18 × 14 × 12 cm) filled with soil and were maintained at ~23 °C and 40% relative humidity with a 16 h photoperiod. They were fed weekly with mealworms (*Tenebrio molitor*) and 20% sucrose solution. We examined the collected ants under a VHX-5000 microscope (Keyence, Neu-Isenburg, Germany) to verify the species using an identification key [62].

### 5.2. Collection of Venom and Venom Glands

We developed a noninvasive venom-sampling protocol in which ants were gently lifted by the thorax using watchmakers’ forceps, allowing their abdomen to be submerged in 500 µL methanol in a 1.5-mL Eppendorf tube for 30 s. This induced the animals to release venom into the solvent. The ants were then transferred to a small plastic container and their venom glands were dissected under a light microscope. The venom glands and remaining body tissue were stored separately in TRIzol reagent (Invitrogen, Carlsbad, CA, USA). We collected samples from 27 ants in each colony of both species. Samples from the same colony were pooled and stored at –80 °C.

### 5.3. RNA Extraction and Sequencing

Total RNA was extracted in TRIzol reagent according to the manufacturer’s instructions and was then treated with Turbo DNase (Thermo Fisher Scientific, Waltham, MA, USA) and RNA Clean and Concentrator 5 (Zymo Research, Irvine, CA, USA). Transcriptome library preparation and sequencing were outsourced to Macrogen (Seoul, Korea). Libraries were prepared using the TruSeq stranded mRNA kit including poly(A) RNA selection. Samples were sequenced on an Illumina NovaSeq system to produce 151 bp paired-end reads. The raw sequence data have been uploaded to the NCBI database (Bio Project PRJNA807911).

### 5.4. Transcriptome Analysis

Read quality was controlled using FastQC v0.11.9 [63]. We removed sequencing adapters and poly(G) tails, and performed quality trimming with cutadapt v2.10 [64]. All RNA-Seq data from the venom glands and remaining body tissues was used for de novo transcriptome assembly by applying a multiple assembler strategy comprising Trinity v2.11.0 [65] with HPC GridRunner v1.0.2 [66] and a minimum assembled contig length of 30, rnaSPAdes v3.14.1 [67] and one additional rnaSPAdes assembly, based on reads corrected with Rcorrector v1.0.4 [68]. Both rnaSPAdes assemblies were run with *k*-mer sizes of 21, 33 and 55. Assemblies from all three approaches were concatenated and clustered using CD-HIT-EST v4.8.1 [69] with a sequence identity of 1. Further steps were applied to the clustered de novo transcriptome. To verify the assembly, we ran BUSCO v4.1.4 [70] with lineage dataset hymenoptera_odb10 (2020-08-05). Trimmed reads were mapped with HISAT2 v2.2.1 [71] against the assembly and counted with StringTie v2.1.6 [72] to calculate TPM values. Gene expression for each species was calculated using the mean TPM value of both colonies for each species. We used TransDecoder v5.5.0 [65] to identify ORFs with a minimum protein length of 10 amino acids for the proteomics peptide search. We also used SAMtools [73] for data conversions.

A blastp v2.10.1 [74] search against UniProtKB/Swiss-Prot v2020_06 [75], VenomZone [76] (downloaded on 11 January 2021) and UniProtKB/Swiss-Prot Tox-Prot [77] (downloaded on 15 January 2021) was performed on confirmed ORFs to identify venom toxins. The E-value was set to a maximum of 1 × 10^−3^ and max_target_seqs was set to the size of the query database. For each BLAST hit, we calculated the coverage of query and subject, and similarity with the BLOSUM62 matrix [78] was assessed using BioPython v1.77 [79]. Results were sorted by similarity, query and subject coverage descending for each venom candidate. The resulting top BLAST hit was used for further analysis. We screened InterProScan v5.52-86.0 [80] with all included databases. Putative venom components were assigned to the corresponding venom family.

We manually verified the identity of venom components in comparative alignments for each venom protein family using MAFFT v7.490 [81], Jalview v2.11.1.4 [82], FastTree v2.1.11 [83] and iTol v6 [84] with predictions from SignalP v6.0g [85]. Comparison sequences were collected from VenomZone (release October 2021), UniProtKB/Swiss-Prot v2021_04, and UniProtKB/TrEMBL v2021_04. We also applied alignment seeds available from Pfam v35.0 [86]. Program calls with parameters used are listed in Table S5.

### 5.5. Species Identification by CO1 Sequence Analysis

Overrepresented sequences identified with FastQC were used to extract the corresponding reads from the raw sequence data. These were transferred to one FASTA file per species along with their corresponding mates, without preserving the mate–pair relationship of the reads. Sequences were then dereplicated using VSEARCH v2.15.1 [87] with derep_prefix and used as a query search (blastn v2.10.1) with default settings and E-value < 1 × 10^−3^ against available Formicidae *CO1* sequences in BOLD [88] (downloaded on 30 November 2020). All hits with 100% identity and 100% query coverage were kept, including multiple matches per read. Overrepresented sequences are listed in Supplementary Table S1.

### 5.6. Proteomics

Our bottom–up proteomics strategy involved a mass spectrometry protocol already applied to animal venoms [48,89]. Briefly, we dissolved 10 μg of sample material in 25 mM ammonium bicarbonate containing 0.6 nM ProteasMax. We added 5 mM DTT and incubated for 30 min at 50 °C to complete the disulfide reduction, followed by modification with 10 mM iodacetamide for 30 min at 24 °C. After quenching the reaction with excess cysteine, we added a 50:1 ratio of trypsin and digested the venom for 16 h at 37 °C. After stopping the reaction by adding 1% trifluoroacetic acid, we purified the sample using a C18-ZipTip (Merck-Millipore, Burlington, MA, USA), dried it under vacuum and redissolved the material in 10 μL 0.1% trifluoroacetic acid.

We separated the peptides on an UltiMate 3000RSLCnano device (Thermo Fisher Scientific) then injected 1 μg of the sample material into a 50 cm μPAC C18 column (Pharma Fluidics, Thermo Fisher Scientific) in 0.1% formic acid at 35 °C. The peptides were eluted in a linear gradient of 3–44% acetonitrile over 240 min. The column was then washed at a flow rate of 300 nL/min with 72% acetonitrile. The separated peptides were injected into an Orbitrap Eclipse Tribrid MS (Thermo Fisher Scientific) in positive ionization mode with the spray voltage set to 1.5 kV and a source temperature of 250 °C achieved using a TriVersa NanoMate (Advion BioSciences, Ithaca, NY, USA). We scanned the samples in data-independent acquisition mode with the following settings: scanning time = 3 s, *m/z* range = 375–1500 and resolution = 120,000. Auto-gain control was set to standard with a maximum injection time of 50 ms. The most intense ions in each cycle with a threshold ion count > 50,000 and charge states of 2–7 were selected with an isolation window of 1.6 *m/z* for higher-energy collisional dissociation (normalized collision energy = 30%). Fragment ion spectra were acquired in the linear ion trap with a rapid scan rate and normal mass range. The maximum injection time was set to 100 ms and selected precursor ions were excluded for 15 s post-fragmentation.

We used Xcalibur v4.3.73.11 and Proteome Discoverer v2.4.0.305 (both from Thermo Fisher Scientific) for data acquisition and analysis. Proteins were identified using Mascot v2.6.2 [90] by searching against the transcriptome sequences using the following settings: precursor ion mass tolerance = 10 ppm, carbamidomethylation as a global modification, methionine oxidation as a variable modification, and one missed cleavage allowed. Fragment ion mass tolerance in the linear ion trap for MS^2^ detection was set to 0.8 Da and the false discovery rate was limited to 0.01 using a decoy database. For qualitative analysis, we only considered proteins that were identified with a Mascot score of at least 30 and at least two verified peptides. A comprehensive list of all venom components identified with confidence, and their characteristics and annotations, are provided in the Supplementary Materials. Proteomic raw data have been uploaded to PRIDE (PXD033537).

### 5.7. Analysis of EGF-Like Toxins

The *M. ruginodis* EGF-like toxins were used as blastp v2.11.0 queries against the unfiltered TransDecoder output from both species (E-value < 10, word size = 3). We kept only those hits with a query coverage of 100% which led to a resulting E-value < 2.47 × 10^−24^. Then, we manually dereplicated the resulting sequences and renamed the unique ones. We adopted the Touchard nomenclature [10], which is modified from the King nomenclature for arthropod venom peptides [91]. Given that both species we analyzed were myrmicine ants, we named the EGF-like toxins myrmicitoxins (MYRTX), and assigned the prefix “U”, which is used to designate toxins lacking a known molecular target.

SignalP slow–sequential mode for eukarya was used to predict signal peptides. MAFFT and Jalview were used to analyze and confirm our findings compared to the known EGF-like toxins of *Manica rubida* (UniProtKB A0A6G9KJM3) and *Myrmecia gulosa* (UniProtKB P0DSL4). We adjusted the SignalP results (*M. rubra* and *M. ruginodis*) after inspecting the multiple sequence alignment to increase the signal peptide length from 28 to 30 amino acids. Molecular weights and isoelectric points (pI) were calculated using Bio Python v1.79.

For phylogenetic analysis, sequences of all known and herein identified EGF-like toxins were aligned using MAFFT v7.505 FFT-NS-2 at default parameters. We then constructed a maximum likelihood tree with ultrafast bootstrap [92] (based on 20,000 replicates) implemented in IQ-TREE v2.1.2 [93] with ModelFinder [94] enabled (FLU + G4 model). The resulting tree was visualized on iTOL. A blastp v2.11.0 search was performed against a preformatted NCBI nonredundant protein sequence database (downloaded with BLAST script update_blastdb.pl nr on 6 March 2022) with default settings (E-value < 10, word_size = 3, max_target_seqs = 100) to recover similarities between EGF-like toxins and known EGF hormones. The top 100 hits were sorted by similarity to the query sequence and the most similar empirically verified hit with a similar sequence length was used to hypothesize similarity between the toxin and hormone (Supplementary Table S4).

## Figures and Tables

**Table 1:** Characteristics of EGF-like toxins identified in *M. rubra* and *M. ruginodis* venom glands. The lengths of precursor and mature toxin sequences are shown in amino acids, where # denotes the number of AAs, alongside the predicted molecular weights (MW) and isoelectric points (pI) of the mature toxins. Toxins marked with * were detected by proteomic analysis.

Species	Toxin	# AA Precursor	# AA Mature Toxin	MW (kDa)	pI (pH)
*M. rubra*	U-MYRTX-Mrub1a	79	49	5.4	5.29
	U-MYRTX-Mrub1b	86	56	6.0	4.80
	U-MYRTX-Mrub1c	76	46	5.2	4.59
	U-MYRTX-Mrub1d	81	51	5.7	5.78
*M. ruginodis*	U-MYRTX-Mrug1a *	79	49	5.4	5.41
	U-MYRTX-Mrug1b *	79	49	5.4	5.41
	U-MYRTX-Mrug1c	86	56	6.0	4.80
	U-MYRTX-Mrug1d	81	51	5.6	4.72
	U-MYRTX-Mrug1e	79	49	5.3	4.76

**Table 2:** List of ant EGF-like toxins and closely related EGF hormones sorted by clade. The top BLAST hit according to our criteria is shown along with its similarity (percentage) to each toxin. Toxins marked with an * were discovered in this study.

Toxin	BLAST Result ID	Similarity (%)	Clade	EGF-Type
ECTX(02)-Rm1a	RXN00400.1	48.0	ECTX-clade	Vertebrate betacellulin-like
ECTX(02)-Rm1b	RXN00400.1	46.0	ECTX-clade	Vertebrate betacellulin-like
ECTX(02)-Rm1c	XP_032961198.1	50.0	ECTX-clade	Vertebrate betacellulin-like
ECTX(02)-Rm1d	XP_042333623.1	46.3	ECTX-clade	Vertebrate epiregulin-like
ECTX(02)-Rm1e	XP_036083747.1	41.4	ECTX-clade	Vertebrate epiregulin-like
MIITX(02)-Mc1a	XP_041957410.1	62.2	MIICTX-clade	HBEGF-like
U-MIITX(02)-Mg1a	XP_038648772.1	65.8	MIICTX-clade	HBEGF-like
U-MYRTX-Mrub1a *	XP_041634697.1	57.1	MYRTX-clade A	Vertebrate TGF-like
U-MYRTX-Mrub1d *	XP_041634697.1	57.1	MYRTX-clade A	Vertebrate TGF-like
U-MYRTX-Mrug1a *	XP_041634697.1	55.3	MYRTX-clade A	Vertebrate TGF-like
U-MYRTX-Mrug1b *	XP_041634697.1	51.1	MYRTX-clade A	Vertebrate TGF-like
U-MYRTX-Mrug1d *	XP_041634697.1	57.1	MYRTX-clade A	Vertebrate TGF-like
U-MYRTX-Mrug1e *	XP_041634697.1	57.1	MYRTX-clade A	Vertebrate TGF-like
U-MYRTX-Mrub1c *	XP_019908008.1	58.8	MYRTX-clade B	Vertebrate epiregulin-like
U-MYRTX-Mrub1b *	XP_016916180.1	63.0	MYRTX-clade C	Insect Spitz-like
U-MYRTX-Mrug1c *	XP_016916180.1	63.0	MYRTX-clade C	Insect Spitz-like
U18-MYRTX-Mri1a	XP_016916180.1	63.0	MYRTX-clade C	Insect Spitz-like

## Data Availability

Raw proteomic and transcriptomic data are publicly available. Raw proteomic data have been uploaded to PRIDE (PXD033537). Raw transcriptomic data have been uploaded to the NCBI database (BioProject PRJNA807911).

## Supplementary Materials

The following supporting information can be downloaded at: https://www.mdpi.com/article/10.3390/toxins14050358/s1. Supplementary Table S1: Overrepresented sequences reported in FastQC during quality control, Supplementary Table S2: Results of CO1 sequence analysis, Supplementary Table S3: Comprehensive list of venom components, Supplementary Table S4: BLAST results of EGF-like toxins search, Supplementary Table S5: Program calls with parameters, Supplementary Data S01-S11: Resulting alignments from MAFFT runs used to validate the venom families as FASTA files, Data S12: Tree of EGF-like toxins used for Figure 4 in NEWICK format. Listing S1. Shell script to prepare the necessary GTF-file for the program call of StringTie. Listing S2. Python script to calculate molecular weights and isoelectrical points.
