# Supplementary material for: Venomics of the Central European Myrmicine Ants Myrmica rubra and Myrmica ruginodis
Source: Toxins (Basel). 2022 May 21;14(5):358. doi: 10.3390/toxins14050358 (PMC9147725; doi:10.3390/toxins14050358)
Supplement: Supplementary file 1 [file toxins-14-00358-s001.zip › TableS1-S2_CO1_analysis.pdf]

Table S1. Overrepresented sequences reported in FastQC during quality control. These sequences were used to collect raw read sequences for *CO1* sequence analysis.

**Species**	**Overrepresented sequence**
M. rubra	CTCCATTTAAAAGACAACTGATTATGCTACCTTAGCACAGTCAATTTACT<br>CTCCCAACTAAATATAAATTCATAAGTTAAAAAATCTAATTAAATCTTAT<br>CTTAATCACAATTAAGATTTTTAACTTAATAACAAAATTCAAATTTTAAT<br>CTTTCGTACAAATATATTTATTTTTATTAAAGATAGAAACCGATCTGGCT<br>GTCCAATCTAGTAGTGCTGAGCCCAAGTTTTTCGTCGATGGAATGACTTT<br>GTCCTTTCGTACAAATATATTTATTTTTATTAAAGATAGAAACCGATCTG
M. ruginodis	CTCCATTTAAAAGACAACTGATTATGCTACCTTAGCACAGTCAATTTACT<br>CTCCATTTAATTTTTATCTTAATTCAACATCGAGGTCGCAATCATTTTTA<br>CTCCCAACTAAATATAAATTTATAAGTTATAAAAATCTAATTAAATCTTA<br>CTTTCGTACAAATATATTTATTTTTATTAAAGATAGAAACCGATCTGGCT<br>GTCCAATCTAGTAGTGCTGAGCCCAAGTTTTTCGTCGATGGAATGACTTT<br>GTCCTTTCGTACAAATATATTTATTTTTATTAAAGATAGAAACCGATCTG

Table S2. Results of *CO1* sequence analysis. We used overrepresented sequences to extract all corresponding raw reads with their mates, dereplicated them and ran a BLAST search against BOLD. Only hits with 100% identity and query coverage were kept. One read could match more than one record in BOLD.

	Myrmica rubra	***Myrmica ruginodis***
Reads	634,472	1,328,614
Unique reads	40,978	98,119
Unique reads after BLAST search and filtering	1,045	28,119
Matching BOLD records	21 Formicidae<br>160 *Myrmica*<br>2,343 *Myrmica rubra*	13 Formicidae<br>43 *Myrmica*<br>858 *Myrmica ruginodi*s
