# Supplementary material for: Venomics of the Central European Myrmicine Ants Myrmica rubra and Myrmica ruginodis
Source: Toxins (Basel). 2022 May 21;14(5):358. doi: 10.3390/toxins14050358 (PMC9147725; doi:10.3390/toxins14050358)
Supplement: Supplementary file 1 [file toxins-14-00358-s001.zip › TableS5_Parameters_used.pdf]

Table S5. Tools used during the presented study arranged in alphabetically order. Generic parts are marked with curly brackets.

<table>
<tr><th>Tool used and comments</th><th>Version</th><th>Program call with parameters</th></tr>
<tr><td>blastn

(CO1 analysis against BOLD data)</td><td>v2.10.1</td><td>makeblastdb -in {input_bold} \
-dbtype nucl \
-parse_seqids \
-title {title} \
-out {output}

blastn \
-evalue 1e-3 \
-num_threads 20 \
-db {input_bold} \
-query {input} \
-out {output} \
-outfmt "6 std slen qlen ppos qcovhsp qcovs sseq qseq" \
-task "megablast"</td></tr>
<tr><td>blastp

(Tox-Prot, VenomZone, Swiss-Prot, each as query)</td><td>v2.10.1</td><td>cat {input} | \
makeblastdb -in - \
-dbtype nucl \
-out {output_prefix} \
-title {title}

NUM_SEQS=$(blastdbcmd -db {assembly_as_db} -info | grep -oP "[\d,]+(?= sequences)" | tr -d ",")

blastp \
-query "{db_as_query}" \
-db {assembly_as_db} \
-max_target_seqs $NUM_SEQS \
-outfmt "6 std slen qlen ppos qcovhsp qcovs sseq qseq" \
-evalue 1e-3 \
-num_threads 2 \
-word_size 2 \
-out {output}</td></tr>
<tr><td>blastp

(candidates for final alignments for venome serine protease)</td><td>v2.11.0</td><td>makeblastdb -in {input_uniprot_trembl} \
-dbtype prot \
-out {output}

blastp \
-query {input_query} \
-db {input_uniprot_trembl} \
-out {output} \
-outfmt "6 std slen qlen ppos qcovhsp qcovs sseq qseq sgi stitle staxids sscinames scomnames sblastnames sskingdoms salltitles" \
-evalue 10 \
-word_size 3 \
-matrix BLOSUM62 \
-max_target_seqs 500 \
-seg 'no' \
-num_threads 18</td></tr>
<tr><td>blastp

(EGF-like toxins in TransDecoder-</td><td>v2.11.0</td><td>makeblastdb \
-in {input_transdecoder} \
-dbtype prot \</td></tr>
</table>

data and in NCBI nr database)		-out {output}<br><br>blastp \<br>-query {input} \<br>-out {output} \<br>-outfmt "6 std slen qlen ppos qcovhsp qcovs sseq qseq" \<br>-evalue 10 \<br>-word_size 3 \<br>-matrix BLOSUM62 \<br>-max_target_seqs 500 \<br>-seg 'no' \<br>-num_threads 8<br><br><br>update_blastdb.pl nr<br>update_blastdb.pl taxdb<br><br>blastp \<br>query {input_egf_like} \<br>-db {input_nr} \<br>-out {output} \<br>-outfmt "6 std slen qlen ppos qcovhsp qcovs sseq qseq sgi stitle staxids sscinames scomnames sblastnames sskingdoms salltitles" \<br>-evalue 10 \<br>-word_size 3 \<br>-matrix BLOSUM62 \<br>-max_target_seqs 100 \<br>-seg 'no' \<br>-num_threads 8
BUSCO	v4.1.4	busco \<br>--force \<br>--mode tran \<br>--in {input} \<br>--out {output_prefix} \<br>--out_path {output_dir} \<br>--lineage_dataset hymenoptera_odb10.2020-08-05/hymenoptera_odb10 \<br>--offline \<br>--cpu 16
CD-HIT-EST	v4.8.1	cd-hit-est \<br>-i {input} \<br>-o {output} \<br>-c 1 \<br>-n 11 \<br>-p 1 \<br>-g 1 \<br>-M 64000 \<br>-T 32 \<br>-d 40
cutadapt	v2.10	cutadapt \<br>--pair-filter=any \<br>--nextseq-trim=15 \<br>-a "AGATCGGAAGAGCACACGTCTGAACTCCAGTCA" \<br>-A "AGATCGGAAGAGCGTCGTGTAGGGAAAGAGTGT" \<br>--max-n 0 \<br>--minimum-length 15 \

		--too-short-output {output_shortr1} \ --too-short-paired-output {output_shortr2} \ --discard-casava -o {output_r1} \ --paired-output {output_r2} {input} \ --cores=32
HISAT2	v2.2.1	hisat2-build \ {input} \ {output_prefix} \ -p 20 <br>hisat2 \ -x {input} \ --rna-strandness RF \ -1 {input_r1} \ -2 {input_r2} \ -p 20 \ -q \ -S {output_sam} \ --all
InterProScan	v55.52-86.0	interproscan.sh \ --input {input} \ --output-file-base {output_prefix} \ --formats TSV,XML,JSON,GFF3 \ --disable-precalc \ --goterms \ --iprlookup \ --pathways \ --seqtype p \ --tempdir {tempdir} \ --appl CDD,Coils,Gene3D,Hamap,MobiDBLite,PANTHER,Pfam,Phobius,PIRSF,PRINTS,ProSitePatterns,ProSiteProfiles,SFLD,SMART,SUPERFAMILY,SignalP_EUK,SignalP_GRAM_NEGATIVE,SignalP_GRAM_POSITIVE,TIGRFAM,TMHMM \ --cpu 8
IQ-TREE	v2.1.2	iqtree \ -T 3 \ -s {input} \ -B 20000 \ --prefix {output_prefix}
MAFFT <br>(all alignments)	v7.490	zcat {input} | \ mafft \ --localpair \ --maxiterate 1000 \ --thread 8 \ --reorder - > {output}
MAFFT <br>(final alignments for venome serine protease)	v7.490	mafft \ --maxiterate 1000 \ --localpair \ --thread 4 \ {input} > {output}
MAFFT <br>(Fig. 4, EGF-like toxins)	v7.505	mafft \ --retree 2 \ --reorder \ {input} > {output}

Rcorrector	v1.0.4	run_rcorrector.pl \ -t 32 \ -1 {input_left} \ -2 {input_right} \ -od {outputdir}
rnaSPAdes	v3.14.1	spades.py \ --rna \ --ss-rf \ -k 21,33,55 \ --threads 32 \ --memory 320 \ -o {output_dir} \ {input_left} \ {input_right}
SAMtools	v1.11	samtools sort \ -@ 4 \ -m 16384 \ -l 9 \ -o {output_bam_sorted} \ {input}<br><br>samtools index \ -@ 4 \ -b \ {input_bam_sorted} \ {output_bam_indexed}<br><br>samtools stats \ {input_bam_sorted} \ -@ 4 > {output_bam_stats}<br><br>plot-bamstats \ -p {output_prefix} \ {input_bam_stats}
SignalP	v6.0g	Signalp6 \ --fastafile {input} \ --organism eukarya \ --output_dir {output_dir} \ --format all \ --mode slow-sequential
StringTie	v2.1.4	stringtie \ -o {output_transcripts} \ -p 4 \ -G {input_gtf} \ -A {output_gene_abundance} \ -C {output_coverage} \ -b {outputdir_ballgown} \ -e {input_bam}<br><br>prepDE.py \ -i {outputdir_ballgown} \ -g {output_gene_count_matrix} \ -t {output_transcript_count_matrix} \ -l 145
TransDecoder	v5.5.0	TransDecoder.LongOrfs \

		-m 10 \<br>-t {input} \<br>--output_dir {output_dir}
Trinity	v2.11.0	Trinity \<br>--grid_exec "HPCRUNNER" \<br>--min_contig_length 30 \<br>--SS_lib_type RF \<br>--left {input_left} \<br>--right {input_right} \<br>--seqType fq \<br>--max_memory 200G \<br>--bflyCalculateCPU \<br>--CPU 10 \<br>--output {output_dir} \<br>--full_cleanup \<br>--normalize_max_read_cov 50 \<br>--NO_SEQTK
VSEARCH	v2.15.1	vsearch \<br>--derep_prefix \<br>{input} \<br>--output {output}

Listing S1. Shell script to prepare the necessary GTF-file for the program call of StringTie.

```
for i in {input_bam_sorted}
do
   samtools view -H $i
done | \
   sort | \
   uniq | \
   perl -ne '
      if ($_ =~ /^\@SQ\s+SN:(\S+)\s+LN:(\d+)/) {{
         print join("\t", ($1, ".", "transcript", 1, $2, 0, "+", ".","ID=$1")),"\n";
      }}
   ' > {output}
```

Listing S2. Python script to calculate molecular weights and isoelectrical points.

```
from Bio import SeqIO
from Bio.SeqUtils import molecular_weight
from Bio.SeqUtils.IsoelectricPoint import IsoelectricPoint as IP

fasta = {input_proteinlist}

for seq_record in SeqIO.parse(fasta, "fasta"):
   print(seq_record.id)
   seq = seq_record.seq.rstrip("*")
   print("MW: %0.2f" % molecular_weight(seq, "protein"))
   print("pI: %0.2f" % IP(seq).pi())
```
